# Increased NEFA levels reduce blood Mg^2+^ in hypertriacylglycerolaemic states via direct binding of NEFA to Mg^2+^

**DOI:** 10.1007/s00125-018-4771-3

**Published:** 2018-11-13

**Authors:** Steef Kurstjens, Jeroen H. F. de Baaij, Caro Overmars-Bos, Inge C. L. van den Munckhof, Veronica Garzero, Marijke A. de Vries, Benjamin Burggraaf, Janna A. van Diepen, Niels P. Riksen, Joost H. W. Rutten, Mihai G. Netea, Manuel Castro Cabezas, René J. M. Bindels, Frances M. Ashcroft, Cees J. J. Tack, Joost G. J. Hoenderop

**Affiliations:** 1 Department of Physiology (286), Radboud Institute for Molecular Life Sciences, Radboud university medical center, P. O. Box 9101, 6500 HB Nijmegen, the Netherlands; 2 Department of Physiology, Anatomy and Genetics, University of Oxford, Oxford, UK; 3 Department of Internal Medicine, Radboud university medical center, Nijmegen, the Netherlands; 4 Department of Internal Medicine, Center for Diabetes and Vascular Medicine, Franciscus Gasthuis Rotterdam, Rotterdam, the Netherlands; 5 Department for Genomics & Immunoregulation, Life and Medical Sciences Institute (LIMES), University of Bonn, Bonn, Germany

**Keywords:** Albumin, Hypertriacylglycerolaemia, Hypomagnesaemia, Magnesium, Magnesium deficiency, Non-esterified fatty acid, Obesity, Triacylglycerols

## Abstract

**Aims/hypothesis:**

The blood triacylglycerol level is one of the main determinants of blood Mg^2+^ concentration in individuals with type 2 diabetes. Hypomagnesaemia (blood Mg^2+^ concentration <0.7 mmol/l) has serious consequences as it increases the risk of developing type 2 diabetes and accelerates progression of the disease. This study aimed to determine the mechanism by which triacylglycerol levels affect blood Mg^2+^ concentrations.

**Methods:**

Using samples from 285 overweight individuals (BMI >27 kg/m^2^) who participated in the 300-Obesity study (an observational cross-sectional cohort study, as part of the Human Functional Genetics Projects), we investigated the association between serum Mg^2+^ with laboratory variables, including an extensive lipid profile. In a separate set of studies, hyperlipidaemia was induced in mice and in healthy humans via an oral lipid load, and blood Mg^2+^, triacylglycerol and NEFA concentrations were measured using colourimetric assays. In vitro, NEFAs harvested from albumin were added in increasing concentrations to several Mg^2+^-containing solutions to study the direct interaction between Mg^2+^ and NEFAs.

**Results:**

In the cohort of overweight individuals, serum Mg^2+^ levels were inversely correlated with triacylglycerols incorporated in large VLDL particles (*r* = −0.159, *p* ≤ 0.01). After lipid loading, we observed a postprandial increase in plasma triacylglycerol and NEFA levels and a reciprocal reduction in blood Mg^2+^ concentration both in mice (Δ plasma Mg^2+^ −0.31 mmol/l at 4 h post oral gavage) and in healthy humans (Δ plasma Mg^2+^ −0.07 mmol/l at 6 h post lipid intake). Further, in vitro experiments revealed that the decrease in plasma Mg^2+^ may be explained by direct binding of Mg^2+^ to NEFAs. Moreover, Mg^2+^ was found to bind to albumin in a NEFA-dependent manner, evidenced by the fact that Mg^2+^ did not bind to fatty-acid-free albumin. The NEFA-dependent reduction in the free Mg^2+^ concentration was not affected by the presence of physiological concentrations of other cations.

**Conclusions/interpretation:**

This study shows that elevated NEFA and triacylglycerol levels directly reduce blood Mg^2+^ levels, in part explaining the high prevalence of hypomagnesaemia in metabolic disorders. We show that blood NEFA level affects the free Mg^2+^ concentration, and therefore, our data challenge how the fractional excretion of Mg^2+^ is calculated and interpreted in the clinic.

**Electronic supplementary material:**

The online version of this article (10.1007/s00125-018-4771-3) contains peer-reviewed but unedited supplementary material, which is available to authorised users.

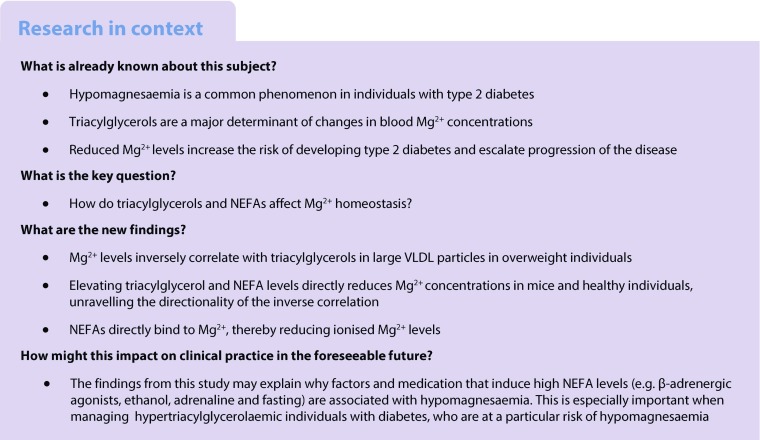



## Introduction

Hypomagnesaemia (blood Mg^2+^ concentration <0.7 mmol/l) is commonly observed in individuals with type 2 diabetes or the metabolic syndrome [1–3] and can result in general complaints such as fatigue, headache and weakness [4, 5]. Low oral Mg^2+^ intake and low blood Mg^2+^ levels not only increase the risk of developing type 2 diabetes but also accelerate disease progression [6–8]. A reduced blood Mg^2+^ is also associated with diabetes-related complications, such as cardiovascular disease and renal failure [9–12].

Blood Mg^2+^ levels are carefully maintained between 0.7 and 1.1 mmol/l by the interplay between intestine, bone and kidney [13]. In blood, approximately 27% of Mg^2+^ is bound to albumin and 8% is complexed to anions, such as phosphate, bicarbonate and citrate, leaving 65% as the free, biologically active form [14]. Although the phenomenon of albumin binding to Mg^2+^ has been known for decades, investigations into the buffering effect of albumin on the regulation of Mg^2+^ homeostasis has been largely neglected [15].

Blood fatty acid and triacylglycerol levels are largely regulated by four organs: the intestine, liver, muscle and adipose tissue. In the postprandial state, the intestine absorbs dietary lipids as fatty acids, which are re-esterified into triacylglycerols and incorporated into chylomicrons that reach metabolically active tissues via the circulation [16]. The liver can also incorporate fatty acids into triacylglycerol and secrete these as VLDL particles; this process is especially important during fasting [17]. Skeletal muscle stores fatty acids in the form of triacylglycerols but also consumes large amounts of fatty acids during exercise [18]. White adipose tissue also stores fatty acids as triacylglycerols, which can be released by lipolysis as NEFAs during a state of energy deprivation [19, 20]. In the blood, these negatively charged NEFAs are bound to carrier proteins, predominantly albumin, via non-polar interactions [21]. In physiological conditions, approximately two NEFA molecules are bound to a single albumin molecule [22]. However, in a state of hypertriacylglycerolaemia, up to seven NEFA molecules are able to bind to albumin, albeit with sequentially lower binding constants [21, 22].

In metabolic diseases, high blood triacylglycerol concentrations are associated with a lower blood Mg^2+^ concentration, but the directionality of this correlation remains unclear [1, 23, 24]. Severe hypomagnesaemia in animals leads to increased blood triacylglycerol levels, possibly by disrupting the function of the enzyme lecithin-cholesterol acyltransferase or by activating lipolysis in adipose tissue [25, 26]. However, whether triacylglycerols can affect Mg^2+^ homeostasis has not yet been investigated.

In this study, we measured serum Mg^2+^ concentrations and plasma lipoprotein concentrations and composition in a cohort of overweight individuals, by use of a metabolomics platform [27]. To further unravel the exact relationship between hypertriacylglycerolaemia and Mg^2+^ levels, we combined a population-based cross-sectional study with in vivo oral lipid loading in both humans and mice, and carried out subsequent investigations in vitro.

## Methods

### 300-Obesity cohort

Three hundred and two individuals, aged 55–80 years, were enrolled in the 300-Obesity cohort study (an observational cross-sectional cohort study within the Human Functional Genetics Projects) at the Radboud university medical center between 2014 and 2016 [28]. The study was carried out in accordance with the Declaration of Helsinki and all participants provided informed consent. All participants had a BMI above 27 kg/m^2^. Individuals with a recent cardiovascular event (myocardial infarction, transient ischaemic attack, stroke <6 months ago), a history of bariatric surgery or bowel resection, inflammatory bowel disease, renal dysfunction or increased bleeding tendency, or who used oral or subcutaneous anticoagulant therapy or thrombocyte aggregation inhibitors (other than acetylsalicylic acid and carbasalate calcium) were excluded. Blood samples were taken in the morning, following an overnight fast. Blood glucose, triacylglycerols, total cholesterol and HDL-cholesterol were measured using standard laboratory procedures (Cobas C8000; Roche Diagnostics, Risch-Rotkreuz, Switzerland). The HOMA-IR was calculated using the standard formula, as published by Matthews et al [29]. High-throughput nuclear magnetic resonance (NMR) metabolomics platform (Nightingale’s Biomarker Analysis Platform, Nightingale Health, Helsinki, Finland) [27] was used for the quantification of 231 lipid and metabolite measures. The metabolites were measured in a single experiment, set-up for the quantification of different metabolite groups. In this article we focus on lipoproteins: total lipid concentrations of 14 lipoprotein subclasses, lipoprotein particles sizes, apolipoproteins and cholesterol. The NMR metabolomics platform used in this study has previously been used in various epidemiological studies [30, 31]. Details of the NMR-based metabolomics experimentation have been described previously [27]. In the present study, serum Mg^2+^ levels were measured in all individuals. However, 17 measurements had substantial duplo errors (large deviations [≥0.05 mmol/l from the average] between duplicate measurements) and, therefore, only 285 individuals were included in the analysis.

### Oral gavage of olive oil in wild-type mice

This animal study was approved by the animal ethics board of the Radboud University Nijmegen (RU DEC 2015-0073) and by the Dutch Central Commission for Animal Experiments (CCD, AVD103002015239). Twelve male C57BL6/J mice (Charles River, Sulzfeld, Germany) were obtained at an age of 9–10 weeks. Mice were acclimatised for 2 weeks in a temperature- and light-controlled room, with six mice per cage (Eurostandard Type III, Tecniplast, Buguggiate, Italy), and were allowed free access to acidified tap water and standard pellet chow (Ssniff Spezialdiäten, Soest, Germany). After the acclimatisation period, mice received experimental chow containing 18.3% protein (wt/wt), 4.1% crude fat (wt/wt), 25.1% starch (wt/wt) and 33.6% sugar (wt/wt) (E15000-04; Ssniff Spezialdiäten). After 2 weeks on the synthetic diet, mice were fasted overnight, from 21:00 to 09:00 hours, before receiving 200 μl intragastric olive oil (extra virgin; Carbonell, Cordoba, Spain) via oral gavage. Blood was drawn via tail-bleed, using chilled sodium-heparin capillary tubes (Praxisdienst, Longuich, Germany) coated with paraoxon (Sigma, St Louis, MO, USA), before the gavage (0 h) and at 1, 2, 4, 6 and 8 h post gavage. The blood was centrifuged at 3000 *g* and plasma was procured.

### Oral gavage of olive oil in a mouse model of diabetes

All procedures for this experiment were conducted in compliance with the UK Animals Scientific Procedures Act (1986) and University of Oxford ethical guidelines. Kir6.2-p.Val59Met mice were generated in our laboratories, as previously published [32]. All mice were 12–16 weeks old. Mice were housed with a maximum of five animals per cage (ventilated), separated by gender and with ad libitum access to food and standard pellet chow, in a 12 h light/dark cycle, at 21°C. In four male and four female C57BL6 mice, expression of a Kir6.2-p.Val59Met transgene was induced using subcutaneous injection of 400 μl tamoxifen (0.02 g/ml corn oil). This inducible mouse model recapitulates the phenotype of neonatal diabetes and these mice develop diabetes due to impaired insulin secretion [32, 33].

Three days after tamoxifen injection, mice were fasted overnight from 17:00 to 09:00 hours. Successful induction of the Kir6.2-p.Val59Met transgene was validated by measuring fasted blood glucose levels using a StatStrip Xpress glucose meter (Nova Biomedical, Waltham, MA, USA). One female mouse did not have elevated fasting glucose levels and was excluded from subsequent analyses. The remaining mice then underwent oral gavage and blood sampling using an identical experimental set up to that described above for C57BL6/J wild-type mice.

### BSA and fatty-acid-free BSA solutions

BSA and fatty-acid-free BSA (FF-BSA) (both Sigma Aldrich) were separately dissolved at several concentrations in a 1 mmol/l MgCl_2_ solution (Merck Millipore, Darmstadt, Germany) or a physiological buffer, both set at pH 7.5 by adding NaOH. The physiological buffer contained the following (all from Merck Millipore unless stated otherwise): 27 mmol/l NaHCO_3_, 112 mmol/l NaCl, 5 mmol/l KCl, 1 mmol/l MgCl_2_, 1 mmol/l Na_2_HPO_4_ (VWR International, Radnor, PA, USA) and 2.5 mmol/l CaCl_2_ dissolved in Milli-Q (Radboud Institute for Molecular Life Sciences, Nijmegen, the Netherlands).

### Increasing NEFA levels in BSA, FBS and MgCl_2_ solutions

To extract endogenous NEFAs, BSA (0.2 g/ml) dissolved in Milli-Q was mixed (1:2 vol./vol.) with ice-cold ethanol–diethyl ether (3:1 vol./vol.). The lipid phase was evaporated overnight at room temperature. To remove trace amounts of BSA, the solution was centrifuged three times with an Amicon 50 kDa filter (Merck Millipore) for 15 min at 2500 *g* to clog the protein in the filter. Extracted NEFAs were added to 250 μl FBS (Biowest, Nuaillé, France), 1 mmol/l MgCl_2_, or 0.5 mmol/l BSA in the amounts of 0, 25, 50, 100, 200, 350, 500 and 700 μl. Dilution factors were accounted for when measuring the concentrations of Mg^2+^ and NEFA.

### Analytical measurements

Protein (Pierce, Thermo Scientific, Waltham, MA, USA), NEFA (WAKO Diagnostics, Delfzijl, the Netherlands), triacylglycerols (Roche Molecular Biochemicals, Indianapolis, IN, USA) and Mg^2+^ (Roche/Hitachi, Tokyo, Japan) concentrations were measured using a spectrophotometric assay according to the manufacturer’s protocols. The Mg^2+^ colourimetric assay was based on a Xylidyl Blue-I method and the absorbance was measured at 600 nm. NEFAs were measured at 546 nm, triacylglycerols at 500 nm and protein at 562 nm on a Bio-Rad Benchmark plus microplate spectrophotometer (Bio-Rad laboratories, Hercules, CA, USA). For inductively coupled plasma mass spectrometry (ICP-MS) analyses, serum samples were dissolved in HNO_3_ (>65%, Sigma) and diluted prior to being subjected to ICP-MS (X-series; Thermo Scientific).

### Oral lipid load in human participants

As part of a study (ClinicalTrials.gov registration no. NCT01967459), which aimed to examine the effect of vitamin D supplementation on postprandial leucocyte activation, 24 female volunteers underwent an oral fat-loading test. The study was approved by the Institutional Review Board of the Franciscus Gasthuis & Vlietland Rotterdam and the regional independent medical ethics committee of the Maasstad Hospital Rotterdam [34]. All participants provided informed consent. Samples from the baseline oral fat load were used in this study to measure serum Mg^2+^, NEFA and triacylglycerol concentrations.

Inclusion criteria were an age above 18 years, a premenopausal status, BMI ≥25 kg/m^2^ and vitamin D deficiency. Exclusion criteria were the use of any kind of medication (except for oral contraceptives), smoking, pregnancy, participation in a clinical study less than 6 months before inclusion and the use of vitamin supplements.

Participants visited the hospital after a 10 h overnight fast and venous blood samples were obtained after an oral fat load (before Vitamin D treatment). Each participant received an oral fat load using fresh cream (Albert Heijn, Zaandam, the Netherlands) at a dose of 50 g of fat per m^2^ body surface calculated by the Mosteller formula [35]. During the oral fat loading, test participants were not allowed to eat or to drink (except water) and they were asked to refrain from physical activity. Venous blood sampling was repeated at 2 h intervals until 8 h after oral fat loading, and serum Mg^2+^ and NEFA levels and plasma triacylglycerol levels were measured (as detailed in the ‘Analytical measurements’ section above). Two individuals were excluded due to insufficient sample availability.

### Statistical analyses

Results are presented as mean ± SEM, unless stated otherwise. Variables of overweight individuals were correlated univariately to serum Mg^2+^ levels using Pearson’s correlation analyses (SPSS for Windows v22.0.0.1, RRID:SCR_002865; IBM, New York, NY, USA). Based on the initial experiment in wild-type mice, the sample size for the experiment in Kir6.2-p.Val59Met mice was calculated using a one-way ANOVA statistic (with Dunnett’s correction for multiple comparison); to detect an effect size of 0.3 (SD 0.13) with a power of 80% and α level of 5%: a total of six mice were required per group. The sample size of the oral lipid (cream) load study in healthy individuals was assessed using a one-way ANOVA (with Dunnett’s correction for multiple comparison); to detect an effect size of 0.1 (SD 0.1) with a power of 80% and an α level of 5%: a total of 23 individuals were required. Significance of Mg^2+^, triacylglycerol and NEFA concentrations at 1, 2, 4, 6 and 8 h compared with those at 0 h was evaluated using a one-way ANOVA with Dunnett’s correction for multiple comparisons. Direct correlations between Mg^2+^ and triacylglycerol or NEFA concentrations were assessed using linear regression analyses. A *p* value of ≤0.05 was considered statistically significant. All statistical analyses were performed using Graphpad Prism v7 (RRID:SCR_002798; Graphpad software, La Jolla, CA, USA).

## Results

### Serum Mg^2+^ levels inversely correlate with blood triacylglycerol levels in overweight individuals

Factors affecting blood Mg^2+^ levels were evaluated in 285 overweight individuals (BMI >27 kg/m^2^) from the 300-Obesity cohort (ESM Table 1). The mean serum Mg^2+^ concentration in this cohort was 0.89 ± 0.09 (SD) mmol/l, with only 2% of the individuals having hypomagnesaemia (serum Mg^2+^ <0.7 mmol/l, see ESM Table 1 and ESM Fig. 1). Despite the fairly healthy serum Mg^2+^ levels in these individuals, serum Mg^2+^ concentrations were inversely correlated with triacylglycerol levels, predominantly those in VLDL particles (Table 1). The serum Mg^2+^ concentration was also inversely correlated with HOMA-IR. As HOMA-IR was strongly correlated with plasma triacylglycerol concentration (ESM Tables 2, 3), we questioned whether insulin resistance modulated the inverse correlation between serum Mg^2+^ and plasma triacylglycerol levels and triacylglycerols in VLDL particles. However, HOMA-IR did not influence these correlations in multivariable regression analyses (ESM Tables 4 and 5).

**Table 1:** Univariate analyses for the correlation of demographics, laboratory measurements and lipoprotein particle concentration with serum Mg^2+^ concentration in overweight individuals from the 300-Obesity cohort

Variable	Correlation coefficient	*p* value	*n*
Demographics			
Sex (M = 0, F = 1)	−0.051	0.391	285
BMI (kg/m^2^)	−0.063	0.291	285
Age (years)	−0.099	0.096	285
Waist circumference (cm)	−0.071	0.231	285
SBP (mmHg)	−0.016	0.791	285
DBP (mmHg)	0.079	0.186	285
Heart rate (beats/min)	−0.083	0.164	282
Laboratory measures			
Triacylglycerols (mmol/l)	−0.159	0.007	284
Glucose (mmol/l)	−0.062	0.299	284
HbA_1c_ (mmol/mol)	−0.032	0.595	284
HOMA-IR	−0.123	0.038	283
Total cholesterol (mmol/l)	0.041	0.495	284
Triacylglycerols (mmol/l)			
in VLDL	−0.158	0.008	284
in LDL	−0.026	0.667	284
in HDL	−0.052	0.380	284
Cholesterol (mmol/l)			
in VLDL	−0.093	0.118	284
in LDL	0.075	0.205	284
HDL	0.116	0.050	284
ApoA1 (g/l)	0.071	0.235	283
ApoB (g/l)	−0.037	0.539	283
Mean lipoprotein diameter (nm)			
VLDL	−0.095	0.111	284
LDL	−0.030	0.612	284
HDL	0.026	0.320	284
Lipoprotein particle concentration (mol/l)			
Chylomicrons and EL-VLDL	−0.170	0.004	284
VL-VLDL	−0.174	0.003	284
L-VLDL	−0.163	0.006	284
M-VLDL	−0.149	0.012	284
S-VLDL	−0.108	0.068	284
VS-VLDL	−0.004	0.942	284
IDL	0.058	0.333	284
L-LDL	0.060	0.313	284
M-LDL	0.059	0.319	284
S-LDL	0.053	0.377	284
VL-HDL	0.020	0.739	284
L-HDL	0.089	0.137	284
M-HDL	0.163	0.006	284
S-HDL	0.179	0.002	284

ApoA1, apolipoprotein A1; ApoB, apolipoprotein B; DBP, diastolic blood pressure; EL-VLDL, extra-large VLDL; F, female; IDL, intermediate-density lipoprotein; L-HDL, large HDL; L-LDL, large LDL; L-VLDL, large VLDL; M, male; M-HDL, medium HDL; M-LDL, medium LDL; M-VLDL, medium VLDL; SBP, systolic blood pressure; S-HDL, small HDL; S-LDL, small LDL; S-VLDL, small VLDL; VL-HDL, very large HDL; VL-VLDL, very large VLDL; VS-VLDL, very small VLDL

To further investigate the relationship between lipoproteins and serum Mg^2+^ level, the composition of lipoprotein particles was investigated and the correlation of these particles with serum Mg^2+^ concentration was analysed (Table 1). The concentration of the larger VLDL particles showed the strongest inverse correlations with serum Mg^2+^ levels (Table 1). Interestingly, the concentration of smaller HDL particles was positively correlated with serum Mg^2+^. There was no correlation between serum Mg^2+^ levels and the concentration of any of the intermediate-density lipoprotein and LDL particles (Table 1). No significant correlation was observed between serum Mg^2+^ and the diameter of the VLDL, LDL or HDL particles (Table 1).

### Increased triacylglycerol levels directly reduce plasma Mg^2+^ concentrations in mice

**Fig. 1:**
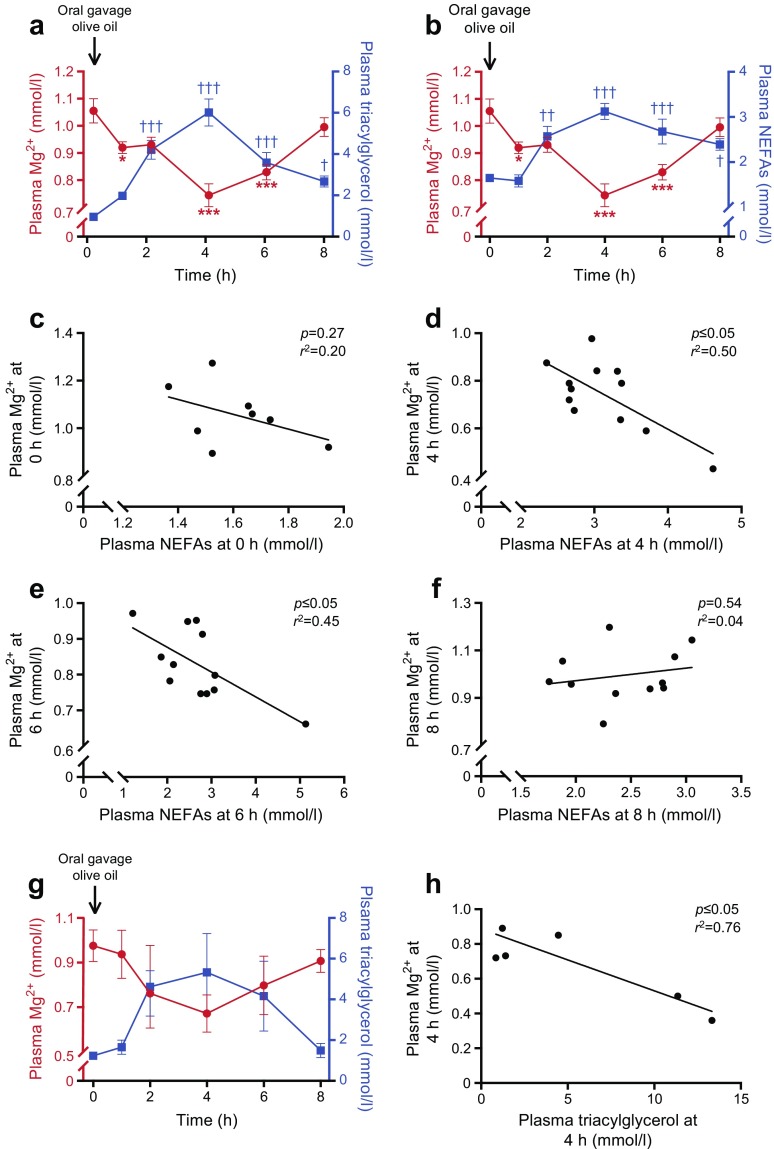
Increased plasma NEFA and triacylglycerol levels directly reduce plasma Mg^2+^ concentration in mice. Mice were given an oral gavage of 200 μl olive oil. (**a**) Plasma Mg^2+^ (red circles) and triacylglycerol (blue squares) and (**b**) plasma Mg^2+^ (red circles,) and NEFA (blue squares) concentrations before (0 h) and at 1, 2, 4, 6 and 8 h post-gavage in wild-type mice (*n* = 12). (**c**–**f**) Linear regression analyses of plasma Mg^2+^ and NEFA concentrations at 0 h (**c**, *n* = 8), 4 h (**d**, *n* = 12), 6 h (**e**, *n* = 12) and 8 h (**f**, *n* = 11) post gavage in wild-type mice, using data from (**b**). Each point represents an individual mouse; several data points are missing due to insufficient sample availability. (**g**) Plasma Mg^2+^ (red circles) and triacylglycerol (blue squares) concentrations before (0 h) and at 1, 2, 4, 6 and 8 h post-gavage in hypoinsulinaemic Kir6.2-p.Val59Met mice (*n* = 7). (**h**) Linear regression analysis of plasma Mg^2+^ and triacylglycerol concentration at 4 h post-gavage, using data shown in (**g**). Each point represents an individual mouse; *n* = 2 mice were excluded due to insufficient sample availability. Data are mean ± SEM. *p ≤ 0.05, ***p ≤ 0.001 vs Mg^2+^ concentrations at 0 h; ^†^*p* ≤ 0.05, ^††^*p* ≤ 0.01, ^†††^*p* ≤ 0.001 vs triacylglycerol or NEFA concentrations at 0 h. Significance was evaluated using a one-way ANOVA with Dunnett’s correction for multiple comparisons

To unravel the underlying mechanism that explains how blood triacylglycerols are associated with blood Mg^2+^ concentrations, mice were subjected to an oral gavage of olive oil following an overnight fast. Plasma triacylglycerols and NEFA levels both peaked at 4 h post-gavage (Fig. 1a, b, Δ plasma Mg^2+^ −0.31 mmol/l at 4 h post-gavage). Interestingly, the plasma Mg^2+^ concentration showed a reciprocal decrease, reaching a nadir at 4 h post-gavage (Fig. 1a, b). At basal levels (0 h), no significant correlation was observed between plasma Mg^2+^ levels and NEFA concentrations (Fig. 1c, *p* = 0.27). However, when plasma NEFA levels increased (at 4 and 6 h), there was a clear inverse correlation between plasma Mg^2+^ and NEFA concentrations (Fig. 1d, e, *p* ≤ 0.05). When plasma NEFA levels decreased and reached a concentration approaching levels at baseline (8 h), there was no longer a significant correlation between plasma NEFA and Mg^2+^ concentrations (Fig. 1f, *p* = 0.54). Similar correlations were observed between plasma Mg^2+^ and triacylglycerol levels (ESM Fig. 2a–d).

Increased blood lipids can enhance glucose-induced insulin secretion [36]. Insulin induces a compartmental shift of Mg^2+^, decreasing the blood concentration of Mg^2+^ and increasing intracellular levels [37]. Hence, to rule out insulin-dependent effects, the same oral gavage experiment was performed in inducible Kir6.2-p.Val59Met mice, which develop hypoinsulinaemia and hyperglycaemia [38]. In these mice, a similar reduction in plasma Mg^2+^ was observed in response to the oral gavage of olive oil as compared with wild-type mice (Fig. 1g). However, while the reduction in the plasma Mg^2+^ concentration was numerically similar to that observed in wild-type mice, it did not reach statistical significance in Kir6.2-p.Val59Met mice due to a high variation between the animals (which was substantially larger than in the initial experiment using wild-type mice). However, a significant inverse correlation between plasma Mg^2+^ and triacylglycerols was still observed in these hypoinsulinaemic mice (Fig. 1h, *p* ≤ 0.05).

### Binding of Mg^2+^ to albumin is NEFA-dependent

**Fig. 2:**
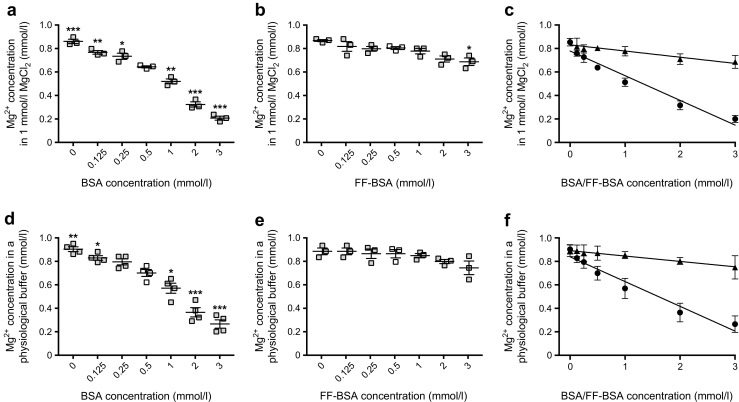
Binding of Mg^2+^ to albumin is NEFA-dependent. (**a**, **b**) The Mg^2+^ concentration in an increasing level of BSA (**a**) or FF-BSA (**b**) dissolved in a MgCl_2_ solution. The white squares indicate a BSA/FF-BSA concentration (0.5 mmol/l) that is near the physiological range; all other data points are shown in grey. (**c**) Linear regression analyses of the Mg^2+^ concentration in increasing levels of BSA (circles; *y* = −0.21*x* + 0.78, *r*^2^ = 0.96, *p* ≤ 0.05) or FF-BSA (triangles; *y* = −0.05*x* + 0.83, *r*^2^ = 0.90, *p* ≤ 0.05), using data shown in (**a**) and (**b**). (**d**, **e**) The Mg^2+^ concentration in an increasing level of BSA (**d**) or FF-BSA (**e**) dissolved in a physiological buffer. The white squares indicate a BSA/FF-BSA concentration (0.5 mmol/l) that is near the physiological range; all other data points are shown in grey. (**f**) Linear regression analyses of the Mg^2+^ concentration in increasing levels of BSA (circles; *y* = −0.21*x* + 0.78, *r*^2^ = 0.96, *p* ≤ 0.05) or FF-BSA (triangles, *y* = −0.05*x* + 0.83, *r*^2^ = 0.90, *p* ≤ 0.05), using data shown in (**d**) and (**e**). Data are mean ± SEM of three replicate experiments. *p ≤ 0.05, **p ≤ 0.01, ***p ≤ 0.001 vs Mg^2+^ concentrations in 0.5 mmol/l BSA/FF-BSA. Significance was evaluated using a one-way ANOVA with Dunnett’s correction for multiple comparisons (**a**, **b**, **d**, **e**) or using a linear regression analysis (**c**, **f**)

As ~30% of Mg^2+^ is bound to albumin, which is the predominant carrier of NEFAs, we investigated whether the binding of Mg^2+^ to albumin is dependent on NEFAs [14]. With increasing concentrations of BSA in MgCl_2_, we found that the Mg^2+^ concentration declined as BSA concentrations increased (Fig. 2a). Mg^2+^ concentrations decreased from 0.85 ± 0.02 mmol/l at 0 mmol/l BSA to 0.64 ± 0.01 mmol/l at a near-physiological concentration of BSA (0.5 mmol/l or 33.25 g/l), a 25% reduction (Fig. 2a). Interestingly, the absence of fatty acid (i.e. the use of FF-BSA instead of BSA in the MgCl_2_ solution) abrogated this effect, in line with the theory that binding of Mg^2+^ to albumin is NEFA-dependent (Fig. 2b). Linear regression analyses showed a significant inverse correlation between Mg^2+^ and BSA concentration (Fig. 2c); the regression constant was approximately four times stronger for BSA than for FF-BSA (Fig. 2c). To exclude the possibility that other cations present in blood compete with the binding of Mg^2+^ to BSA, BSA was also dissolved in a physiological buffer, which mimicked the concentration of other abundant blood electrolytes. This approach resulted in a similar decrease in the Mg^2+^ concentration with increasing BSA concentrations (Fig. 2d). Again, FF-BSA had no significant effect on Mg^2+^ levels (Fig. 2e). In the physiological buffer, BSA and FF-BSA displayed correlations similar to those observed in the MgCl_2_ buffer (Fig. 2f).

### NEFAs directly decrease Mg^2+^ concentrations

**Fig. 3:**
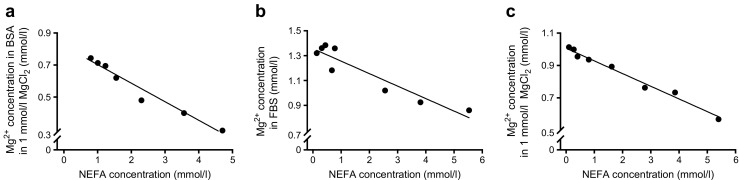
NEFAs directly reduce Mg^2+^ concentrations. Linear regression analyses of Mg^2+^ and NEFA concentration in (**a**) BSA dissolved in 1 mmol/l MgCl_2_ (*y* = −0.12*x* + 0.83, *r*^2^ = 0.97, *p* ≤ 0.05), (**b**) FBS (*y* = −0.10*x* + 1.36, *r*^2^ = 0.90, *p* ≤ 0.05) and (**c**) MgCl_2_ solution (*y* = −0.08*x* + 1.00, *r*^2^ = 0.99, *p* ≤ 0.05). Results from one representative experiment are shown. The experiment was repeated three additional times with similar results

We then set out to modify the binding of Mg^2+^ to albumin by increasing the concentration of NEFA. Elevating the concentration of NEFAs in a BSA–MgCl_2_ solution directly reduced the Mg^2+^ concentration (linear regression: *y* = −0.12*x* + 0.83, *p* ≤ 0.05; Fig. 3a). To resemble the in vivo setting, the experiment was repeated using FBS instead of BSA; increasing NEFA levels in FBS reduced Mg^2+^ concentrations to a similar extent as when BSA was used (linear regression: *y* = −0.10*x* + 1.36, *p* ≤ 0.05; Fig. 3b). Interestingly, the NEFA-induced reduction in Mg^2+^ concentration was protein-independent, as increasing NEFA levels in a MgCl_2_ solution also lowered Mg^2+^ concentration (linear regression: *y* = −0.08*x* + 1.00, *p* ≤ 0.05; Fig. 3c).

### Elevating triacylglycerol levels reduces serum Mg^2+^ concentration in healthy individuals

**Fig. 4:**
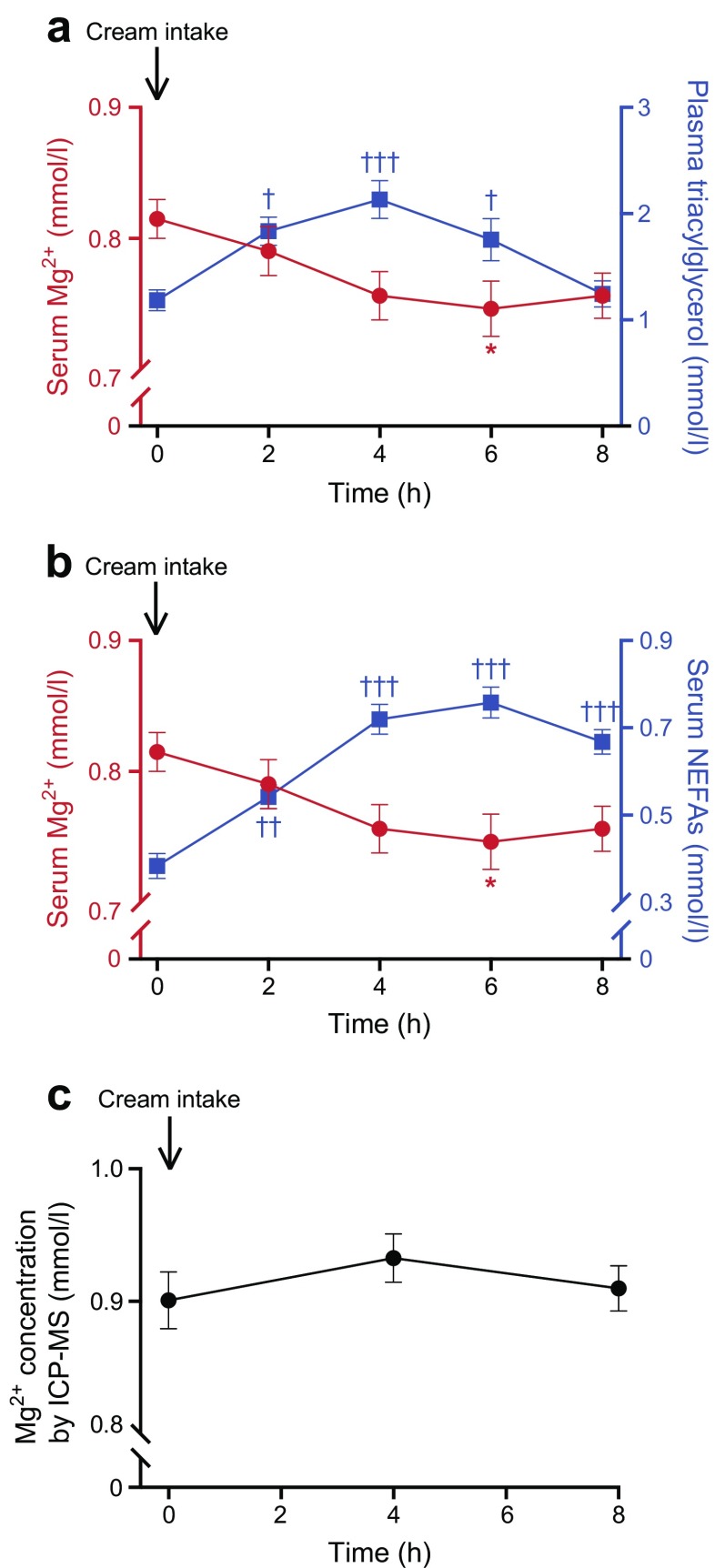
Increased serum NEFA and plasma triacylglycerol levels directly reduce serum Mg^2+^ concentrations in healthy overweight or obese women. Female volunteers underwent an oral fat (cream) loading test. (**a**) Serum Mg^2+^ (red circles) and plasma triacylglycerol (blue squares) and (**b**) serum Mg^2+^ (red circles) and NEFA (blue squares) concentrations before (0 h) and at 1, 2, 4, 6 and 8 h after an oral lipid load in healthy overweight women (*n* = 22). (**c**) Total serum Mg^2+^ levels measured by ICP-MS before (0 h) and 4 and 8 h after oral lipid intake. Data are mean ± SEM.*p ≤ 0.05 vs Mg^2+^ concentrations at 0 h; ^†^*p* ≤ 0.05, ^††^*p* ≤ 0.01, ^†††^*p* ≤ 0.001 vs triacylglycerol or NEFA concentrations at 0 h. Significance was evaluated using a one-way ANOVA with Dunnett’s correction for multiple comparisons

To discover whether triacylglycerols and NEFAs also affect blood Mg^2+^ concentration in humans, we examined samples obtained from 24 healthy female individuals, with a BMI >25 kg/m^2^, who received an oral fat load [34]. Serum NEFA and Mg^2+^ levels and plasma triacylglycerol concentrations were measured over a period of 8 h. Plasma triacylglycerol levels increased significantly, from 1.18 ± 0.10 mmol/l at 0 h to a peak of 2.13 ± 0.18 mmol/l at 4 h (Fig. 4a). Serum NEFA concentrations also significantly increased, from 0.38 ± 0.03 mmol/l at 0 h to a peak of 0.76 ± 0.04 mmol/l at 6 h (Fig. 4b). In accordance with our previous findings, serum Mg^2+^ levels dropped from 0.82 ± 0.01 mmol/l at 0 h to a nadir of 0.75 ± 0.02 mmol/l at 6 h (Fig. 4a, b, Δ plasma Mg^2+^ −0.07 mmol/l at 6 h post-intake). The total serum Mg^2+^ concentration, as measured by ICP-MS, was not affected by fat (cream) intake; hence, these findings suggest that only the free Mg^2+^ levels were affected by lipid loading (Fig. 4c).

## Discussion

Hypomagnesaemia is a common phenomenon in type 2 diabetes. In the current study, we show that high blood NEFA and triacylglycerol concentrations directly reduce blood Mg^2+^ levels. We also show that high NEFA levels bind Mg^2+^, resulting in decreased circulating levels of free Mg^2+^. The conclusions of this study are based on complementary results from in vitro studies and animal and human in vivo studies. First, in a large cohort of overweight individuals, the concentration of triacylglycerols in large VLDL particles was inversely correlated with serum Mg^2+^ concentrations. Second, a dietary lipid load directly reduced blood Mg^2+^ concentration in both mice and humans, independent of insulin action. Third, in vitro, we demonstrated that this phenomenon occurs due to direct binding of NEFA molecules to Mg^2+^. These findings demonstrate that triacylglycerols reduce concentrations of free Mg^2+^ in the blood and consequently place hypertriacylglycerolaemic individuals at risk for Mg^2+^ deficiency.

Here, we demonstrated that increased triacylglycerol and NEFA levels reduce levels of free Mg^2+^(the biologically active form of Mg^2+^) by a direct interaction between negatively charged NEFA molecules and Mg^2+^ ions. This binding was shown to be highly specific for Mg^2+^, since the presence of physiological concentrations of other cations did not affect the interaction between Mg^2+^ and NEFAs. The phenomenon of reduced Mg^2+^ levels as a result of elevated NEFA levels has previously been observed in dogs, although no underlying mechanism was suggested [39]. In addition, previous studies have shown that Ca^2+^ can bind to NEFAs, and that blood Ca^2+^ concentrations are reduced by increasing NEFA levels in individuals [40]. However, in our in vitro experiments, the addition of physiological concentrations of Ca^2+^ did not affect the Mg^2+^–NEFA interaction, indicating a higher affinity of Mg^2+^ compared with Ca^2+^ for binding to NEFAs. This is likely due to the fact that the Mg^2+^ ion has a significantly higher charge density than the Ca^2+^ ion [41].

The findings from this study may explain why certain factors that affect circulating NEFAs are associated with changes in blood Mg^2+^ concentrations. For example, molecules such as β-adrenergic agonists, ethanol and adrenaline (epinephrine), which activate lipolysis and, hence, increase blood NEFA levels, are associated with reduced blood Mg^2+^ levels [1, 42–44]. Indeed, an intravenous infusion of the β-adrenergic agonist terbutaline causes a reduction in serum Mg^2+^ concentration, which is correlated to the elevated concentrations of plasma NEFAs, but not glucose [45]. Although, this does not exclude the possibility of additional potential mechanisms for Mg^2+^ decrease with use of these agents. In addition, prolonged fasting, which induces lipolysis and increases circulating NEFAs, also results in hypomagnesaemia [46].

Approximately 30% of blood Mg^2+^ is bound to albumin [14]. However, our data indicate that the binding of Mg^2+^ to albumin depends on the availability of NEFA. Our findings suggest that direct binding of Mg^2+^ to albumin is minimal, since NEFA-depleted albumin showed little binding to Mg^2+^. To correct for albumin binding, a factor of 0.7 is used when calculating the fractional excretion of Mg^2+^(FEMg): FEMg = [(uMg × sCr)/(sMg × uCr × 0.7)] × 100), where sMg and uMg are the serum and urinary Mg^2+^ levels, respectively, and sCr and uCr are the serum and urinary creatinine levels, respectively [47]. As the large majority of NEFAs in blood are bound to albumin, alterations in NEFA concentrations may be the determining factor in the binding of Mg^2+^ to albumin [22]. This paradigm change questions the current protocol for calculating FEMg. The use of the factor of ‘0.7’ is accurate in physiological conditions but will lead to inaccurate calculations in pathological conditions such as hypertriacylglycerolaemia.

Several limitations need to be considered with regard to this study. Hypertriacylglycerolaemia in mice was achieved using olive oil, while in human volunteers this was done by an oral load of cream. Olive oil contains no Mg^2+^, while the cream used in the human study contains 3.3 mmol/l Mg^2+^, leading to a potential underestimation of the reduction in serum Mg^2+^ in the healthy volunteers. Moreover, in the in vitro experiments, NEFAs extracted from BSA were used to increase NEFA levels in several solutions. However, the yield of this extraction procedure was not equal in each experiment performed, making it difficult to combine data from all experiments. Despite these differences in NEFA yield, the results were similar in all four replicate experiments. Finally, in overweight individuals and in individuals with type 2 diabetes, Mg^2+^ inversely correlates with triacylglycerols [1, 23, 24]. Our in vitro data show direct binding of Mg^2+^ to NEFA molecules, which, in contrast to triacylglycerol molecules, possess a negative charge. It is unlikely that Mg^2+^ binds to uncharged triacylglycerol molecules. However, in humans, blood triacylglycerols and NEFA levels strongly correlate, meaning that most individuals with hypertriacylglycerolaemia also have elevated NEFA levels, which would underlie the inverse correlation between Mg^2+^ and triacylglycerols [48–51].

This study has several strengths. Our data extend from molecule to population and have clinical implications. Moreover, we demonstrated the directionality of the inverse association between triacylglycerols and Mg^2+^, which could explain why hypomagnesaemia is so prominent in diseases such as type 2 diabetes. Our data do not rule out the possibility that changes in Mg^2+^ concentrations could also influence lipid levels.

In conclusion, we show that elevated blood NEFA and triacylglycerol levels directly reduce blood Mg^2+^ concentrations by the binding of Mg^2+^ ions to NEFA molecules. Our data explain the high prevalence of hypomagnesaemia in several metabolic diseases, characterised by elevated triacylglycerol levels [1–3]. In individuals with these diseases, those with hypertriacylglycerolaemia are at particular risk for hypomagnesaemia, and therefore, blood Mg^2+^ levels should be routinely measured and monitored.

## Electronic supplementary material

ESM (PDF 161 kb)

## Data Availability

The data and materials that support the findings of this study are available from the corresponding author upon reasonable request.

## Abbreviations

FF-BSA: Fatty-acid-free BSA
FEMg: Fractional excretion of Mg^2+^
ICP-MS: Inductively coupled plasma mass spectrometry
NMR: Nuclear magnetic resonance
