# Supplementary material for: Increased NEFA levels reduce blood Mg2+ in hypertriacylglycerolaemic states via direct binding of NEFA to Mg2+
Source: Diabetologia. 2018 Nov 13;62(2):311–21. doi: 10.1007/s00125-018-4771-3 (PMC6323097; doi:10.1007/s00125-018-4771-3)
Supplement: Supplementary file 1 — (PDF 161 kb) [file 125_2018_4771_MOESM1_ESM.pdf]

ESM Table 1. Characteristics of the Overweight Individuals from the 300-Obesity Cohort.

Variable	300-Obesity cohort mean ± SD	*n*
**Demographics**		
Sex (m:f, %)	45:55 (m:f)	285
BMI (Kg/m^2)	30.7 ± 3.4	285
Age (years)	67 ± 5	285
Waist circumference (cm)	107 ± 10	285
SBP (mmHg)	130 ± 14	285
DBP (mmHg)	80 ± 9	285
Heart rate (beats/min)	63 ± 10	282
**Laboratory measures**		
Magnesium (mmol/l)	0.89 ± 0.09	285
Triacylglycerols (mmol/l)	1.8 ± 1.0	284
Glucose (mmol/l)	5.7 ± 1.3	284
HOMA-IR	13.7 ± 19.1	283
HbA_{1c} (mmol/mol)	41.8 ± 8.0	284
Total cholesterol (mmol/l)	6.3 ± 1.1	284
Triacylglycerols in VLDL (mmol/l)	1.23 ± 0.72	284
Triacylglycerols in LDL (mmol/l)	0.23 ± 0.07	284
Triacylglycerols in HDL (mmol/l)	0.15 ± 0.04	284
Cholesterol in VLDL (mmol/l)	1.14 ± 0.37	284
Cholesterol in LDL (mmol/l)	2.25 ± 0.64	284
Cholesterol in HDL (mmol/l)	1.31 ± 0.29	284
ApoA1 (g/l)	1.59 ± 0.01	283
ApoB (g/l)	1.19 ± 0.02	283
Mean diameter VLDL particle (nm)	36.6 ± 1.2	284
Mean diameter LDL particle (nm)	23.5 ± 0.1	284
Mean diameter HDL particle (nm)	9.8 ± 0.2	284

ApoA1, apolipoprotein A1; ApoB, apolipoprotein B; BMI, body mass index; DBP, diastolic blood pressure; f, female; HbA1c, glycated hemoglobin; HDL, high-density lipoprotein; HOMA-IR, homeostatic model assessment of insulin resistance; LDL, low-density lipoprotein; m, male; SBP, systolic blood pressure; VLDL, very-low-density lipoprotein.

ESM Table 2. Univariate regression analysis of HOMA-IR, as dependent variable, and plasma triacylglycerols.

**Variable**	**Correlation coefficient**	***p*-value**	***n***
Plasma triacylglycerols (mmol/L)	0.245	0.000	284

ESM Table 3. Univariate regression analysis of HOMA-IR, as dependent variable, and triacylglycerols in VLDL.

**Variable**	**Correlation coefficient**	***p*-value**	***n***
Triacylglycerols in VLDL	0.236	0.000	284

ESM Table 4. Multivariate regression analysis of HOMA-IR and plasma triacylglycerols with the serum Mg^{2+} concentration as dependent variable.

**Variable**	**Correlation coefficient**	***p*-value**	***n***
HOMA-IR	-0.089	0.143	283
Plasma triacylglycerols (mmol/l)	-0.137	0.024	284

HOMA-IR, homeostatic model assessment of insulin resistance

ESM Table 5. Multivariate regression analysis of HOMA-IR and plasma triacylglycerols in VLDL particles with the serum Mg^{2+} concentration as dependent variable.

**Variable**	**Correlation coefficient**	***p*-value**	***n***
HOMA-IR	-0.092	0.128	283
Triacylglycerols in VLDL	-0.137	0.024	284

HOMA-IR, homeostatic model assessment of insulin resistance

ESM1

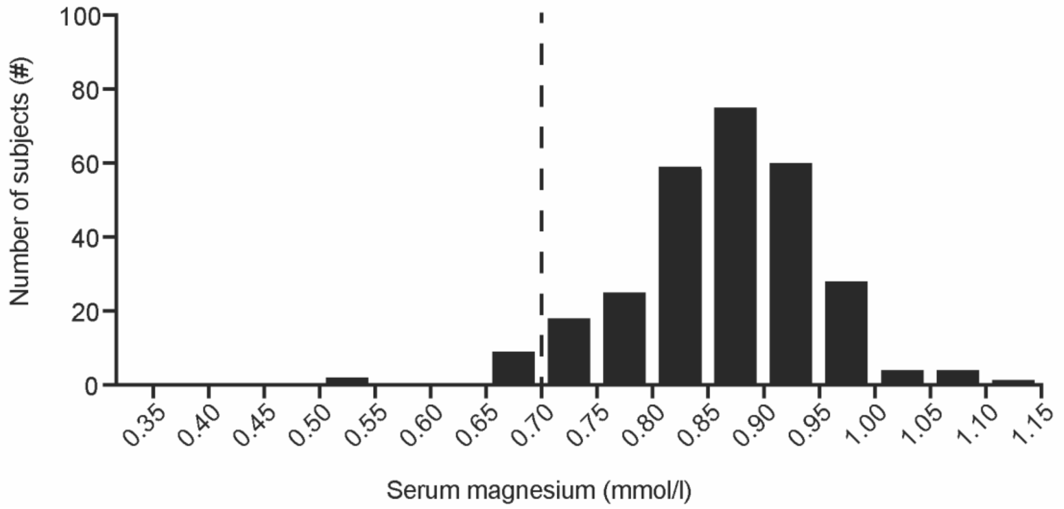

ESM Fig. 1 Serum Mg^{2+} Distribution of the Overweight Subjects of the 300-Obesity Cohort. Distribution of the serum Mg^{2+} concentration of overweight subjects of the 300-Obesity cohort. The dotted vertical line indicates the threshold for hypomagnesemia.

ESM2

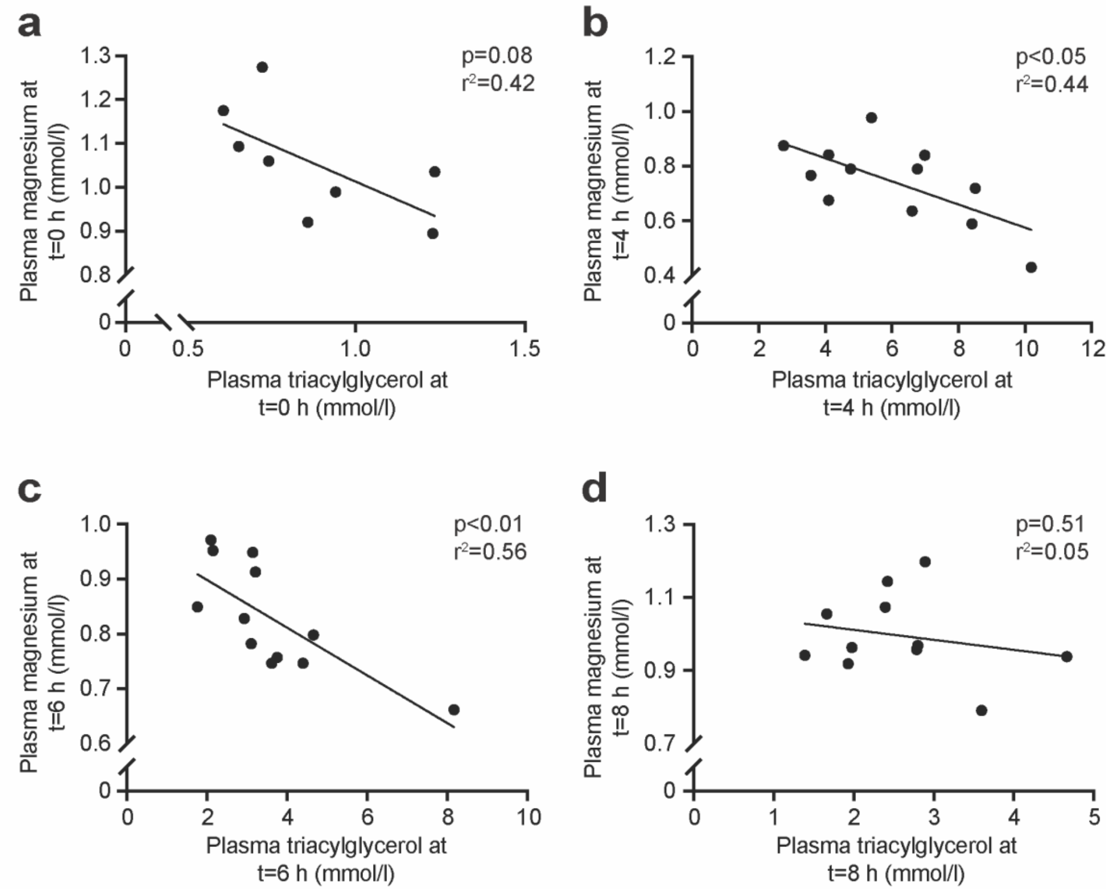

ESM Fig. 2 Plasma Mg^{2+} and triacylglycerol concentrations inversely correlate in hypertriacylglycerolemic states.

(**a-d**) Linear regression analyses between serum Mg^{2+} and triacylglycerol concentrations at t = 0 (**a**, *n*=8), 4 (**b**, *n*=12), 6 (**c**, *n*=12) and 8 (**d**, *n*=11) hours post-gavage in wild-type mice, using data from Figure 1a. Each point represents an individual mouse; several mice were excluded due to insufficient sample availability.
